# Recurrent Rapunzel syndrome in a young woman: Diagnostic pitfalls and surgical strategy

**DOI:** 10.1016/j.ijscr.2025.111672

**Published:** 2025-07-10

**Authors:** Hiba Ben Hassine, Mohamed ridha Zayati, Ghada Naghmouchi, Wided Trimech, Ibtissem Korbi, Faouzi Noomen

**Affiliations:** University of MONASTIR, Faculty of Medicine of Monastir, 5000, Fattouma Bourguiba Hospital, Department of Visceral Surgery, Monastir, Tunisia

**Keywords:** Trichobezoar, Surgical extraction, Diagnostic, Case report

## Abstract

**Introduction and importance:**

Trichobezoars are typically localized to the stomach but can rarely extend into the small bowel, causing a condition known as Rapunzel syndrome. This rare entity primarily affects young females with psychiatric conditions such as trichotillomania and trichophagia. The recurrent nature of this syndrome and its association with psychological disorders necessitate a multidisciplinary approach.

**Presentation of case:**

A 21-year-old female presented to the emergency department with abdominal pain, nausea, and vomiting persisting for four days. On physical examination, she had diffuse abdominal tenderness and a palpable left upper quadrant mass. She had undergone surgery for a trichobezoar in 2015. CT imaging revealed a large heterogeneous intragastric mass extending into the jejunum. Surgical intervention included midline laparotomy, gastrotomy for removal of a large trichobezoar, and enterotomy to extract a second bezoar located 1.4 m from the ileocecal valve, measuring over 30 cm. The patient recovered uneventfully and was referred for psychiatric follow-up. At six months, she remained asymptomatic.

**Discussion:**

Rapunzel syndrome represents an unusual but serious gastrointestinal condition. Diagnosis is often delayed due to its non-specific presentation and rarity. CT imaging is pivotal in detecting the size, shape, and extension of trichobezoars. In this recurrent case, detailed surgical planning allowed for complete removal of both gastric and intestinal masses. Psychiatric evaluation and cognitive-behavioral therapy were initiated postoperatively to prevent recurrence. Key diagnostic challenges include delayed suspicion, misinterpretation of symptoms, and missed psychiatric associations. Surgical techniques must be adapted depending on bezoar size and location, with median laparotomy preferred for extensive disease.

**Conclusion:**

Recurrent Rapunzel syndrome underscores the importance of a holistic, multidisciplinary strategy. Timely radiological diagnosis, careful surgical planning, and sustained psychiatric management are crucial for preventing recurrence. This case reinforces the need for vigilance in young females with behavioral disorders and abdominal complaints.

## Highlights

• Trichobezoars are rare gastric masses composed of ingested hair
• Trichobezoars is Associated with psychiatric disorders like trichotillomania and trichophagia.
• Rapunzel syndrome refers to a trichobezoar extending into the small bowel
• CT imaging is crucial for diagnosis
• Surgery remains the definitive treatment in complicated or extensive cases
• endoscopy is rarely effective for large trichobezoars.
• Psychiatric referral is essential to prevent recurrence

## 1 Introduction

The Rapunzel syndrome, named after the fairy tale character with long tresses, was first documented by Vaughan et al. in 1968 [1]. This syndrome represents a rare form of trichobezoar found in the stomach, extending into the small intestine and causing bowel obstruction. It predominantly affects young females with a history of trichotillomania, trichophagia, or other psychiatric disorders. While often asymptomatic, it can lead to complications such as acute intestinal obstruction. This case report aims to describe the diagnostic challenges and surgical management of a rare and recurrent Rapunzel syndrome. This case report follows the 2025 SCARE guidelines [2].

## 2 Case presentation

**Fig. 1:**
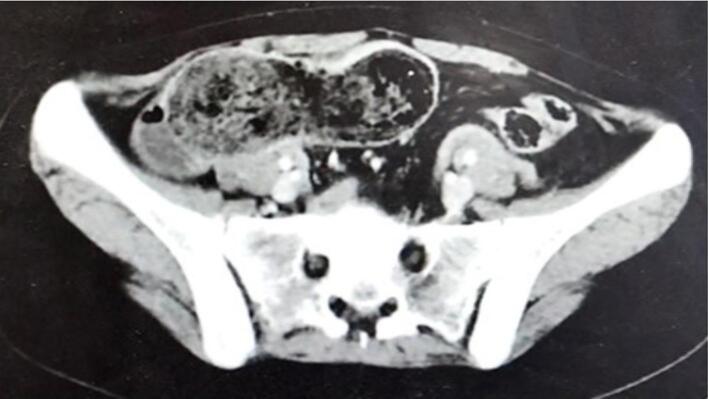
CT scan of the abdomen shows a big bezoar in the stomach and a smaller one in the small intestine.

**Fig. 2:**
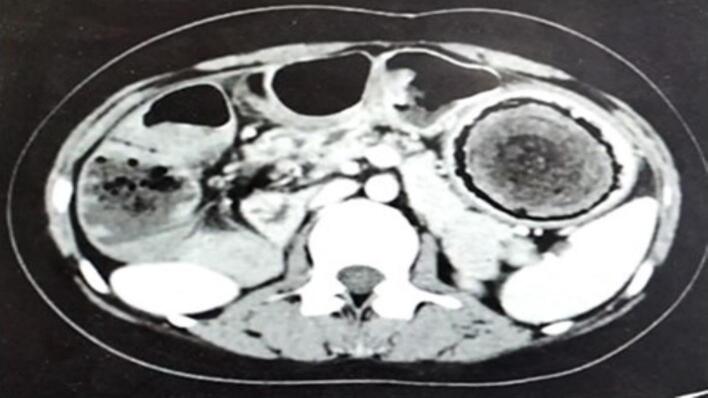
CT scan of the abdomen shows a big bezoar in the stomach and a smaller one in the small intestine.

**Fig. 3:**
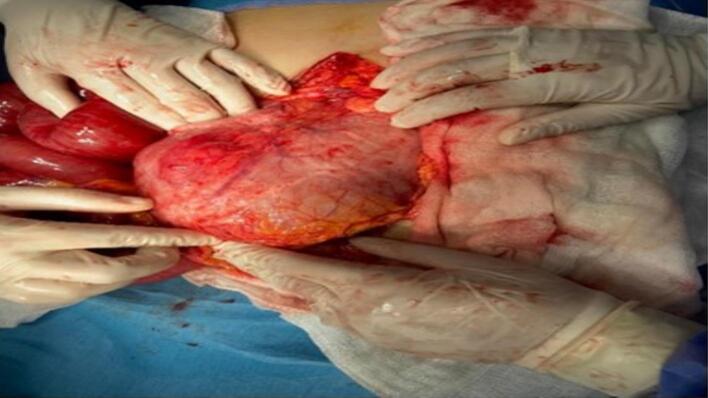
A stomach-shaped trichobezoar surgically removed en bloc by an upper median laparotomy and gastrotomy.

**Fig. 4:**
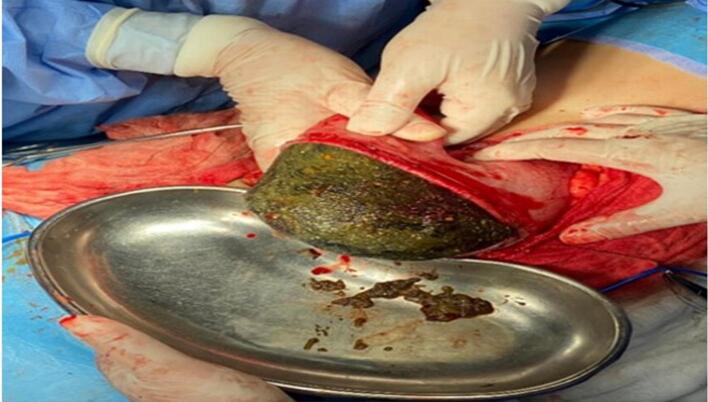
A stomach-shaped trichobezoar surgically removed en bloc by an upper median laparotomy and gastrotomy.

**Fig. 5:**
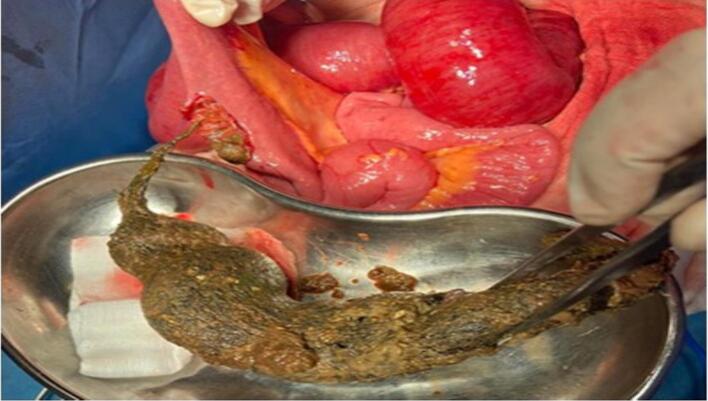
A smaller trichobezoar with a tail was surgically removed en bloc by an upper median laparotomy and ileotomy.

A 21-year-old woman has presented to the emergency with abdominal pain, nausea, and vomiting persisting for four days. During physical examination, diffuse abdominal tenderness was observed with bulging mass in the left hemi abdomen. The patient had a previous history of a trichobezoar surgically removed in 2015, and the patient's mother admitted that she had a habit of pulling out her hair and secretly swallowing it. Laboratory results showed a white blood cell count of 5160 cells/mm^3^ and a C-reactive protein level of 232 mg/L. The patient had microcytic and hypochromic anemia with hemoglobin at 9.5 g/dL. A CT scan revealed gastric distension with heterogeneous contents and intraluminal debris containing air bubbles, forming a mass that matched the shape of the stomach, along with jejunal distention. (Fig. 1, Fig. 2).The diagnosis of a gastroduodenal and intestinal trichobezoar was made. The patient underwent urgent surgery via a midline incision. During the operation, a distended stomach extending to the pelvis (Fig. 3) was found, containing a hard foreign body. A second foreign body extended over 30 cm and was located 1.4 m from the ileocecal valve. A gastrotomy was performed, and the trichobezoar was removed (Fig. 4). An enterotomy away from the inflamed small bowel was made to extract a second trichobezoar (Fig. 5).Postoperative course was uneventful, patient remains asymptomatic and in excellent clinical condition. She was referred to psychiatry for further care. She remains asymptomatic under follow-up and adherent to psychiatric care.

## 3 Discussion

Bezoars form following the ingestion of foreign material that accumulates in the gastrointestinal tract, causing gastric obstruction. Types of bezoars include phytobezoars, trichobezoars, and lactobezoars. This can lead to significant complications, such as dysphagia, abdominal pain, hematemesis, and intestinal obstruction or perforation [3]. Trichobezoars primarily composed of hair, are often seen in patients with a history of gastric surgery in 20 % of cases. These are commonly linked to obsessive-compulsive disorders like trichotillomania and trichophagia, mental retardation, and predominantly affect females [4]. In our case, the patient had no diagnosed psychiatric disorder, but a history of surgery for the same issue six years ago may indicate an undiagnosed psychiatric condition. Trichobezoars are predominantly found in the stomach, but can extend into the small intestine or transverse colon. Rapunzel syndrome is a rare type of trichobezoar. Its definition varies, with some describing it as a gastric trichobezoar with a tail extending to the jejunum or beyond, while others define it by the presence of intestinal obstruction [5].

Trichobezoars may remain asymptomatic or manifest with a variety of clinical presentations. The most common symptoms include an epigastric mass (80 %), abdominal pain (70 %), nausea or vomiting (65 %), fatigue associated with weight loss (38 %), diarrhea or constipation (33 %). The clinical manifestations vary widely due to the size and location of the trichobezoar in the gastrointestinal tract [5].

It can cause various complications, including digestive hemorrhage from parietal ulcerations. Intestinal obstruction by a trichobezoar is rare, occurring in about 10 % of cases, but it is a serious complication that needs prompt recognition and management [5].

Endoscopic exploration reveals the presence of trichobezoar in the stomach. Computed tomography scan show a dilated stomach with a heterogeneous mass, typically containing hair and trapped food surrounded by air and fluid. CT imaging helps determine the size, location, and extent of the obstruction, and identifies any signs of complications such as ischemia or perforation [6].

Multiple treatment options are available for managing trichobezoars, depending on factors like symptoms, size, location, and complications. For small trichobezoars, the initial treatment involves ample fluids to promote transit. If needed, the second-line option is endoscopic removal. Surgical intervention remains the main approach for complicated cases, large masses, or multiple locations. The preferred surgical method is a median laparotomy, which allows for the removal of the gastric trichobezoar through gastrotomy and extraction of any extensions or fragments blocking other parts of the gastrointestinal tract via enterotomies [6]. Our patient underwent surgery with a median incision for gastrotomy and enterotomy. Recurrence of trichobezoars has been documented post-removal. Given the strong association between trichobezoars and underlying psychiatric disorders such as trichotillomania and trichophagia, psychiatric intervention remains a cornerstone in long-term management. Following surgery, our patient was referred to psychiatric services where she began a structured behavioral therapy program. Long-term psychological follow-up was also initiated to monitor for signs of relapse and reinforce coping strategies. Cognitive behavioral therapy in particular has shown promise in reducing recurrence rates by helping patients develop healthier mechanisms for stress and impulse control. This multidisciplinary approach, integrating surgical treatment and comprehensive psychiatric care, is critical to improving outcomes and preventing relapse in patients with Rapunzel syndrome [7]. Active patient engagement in psychiatric interventions is crucial to prevent the recurrence of trichobezoars.

## 4 Conclusion

The Rapunzel syndrome represents an uncommon variant of trichobezoar. While smaller trichobezoars may be managed through endoscopic techniques, the larger trichobezoars associated with Rapunzel syndrome typically require surgical intervention for removal. This condition predominantly affects young females with a history of psychiatric disorders. The diagnosis of Rapunzel syndrome can be confirmed through endoscopic evaluation or computed tomography imaging. Given the high risk of recurrence, it is essential to provide the patient with long-term psychiatric follow-up and management. This multidisciplinary approach, involving both surgical and psychiatric care, is crucial in preventing the recurrence of this challenging condition.

## CRediT authorship contribution statement

All authors participated in the treatment of patients, writing, and approving the manuscript.

Hiba Ben Hassine; Mohamed ridha Zayati, participated in the treatment of the patients and writing the manuscript.

Ghada Naghmouchi, wided Trimech, Ibtissem Korbi, Faouzi Noomen validation of the manuscript.

All the authors approved the manuscript.

## Informed consent

The patient provided written consent for the publication of her clinical details and any identifying imagesList of figures.

## Consent

Written informed consent was obtained from the patient for publication of this case report and accompanying images. A copy of written consent is available for review by the editor-in-chief of this journal upon request.

## Ethical approval

Not applicable.

## Guarantor

Hiba Ben Hassine.

## Declaration of Generative AI and AI-assisted technologies in the writing process

No AI was used in the research and manuscript development.

## Funding

This research did not receive grants from public, commercial, or not-for-profit sectors.

## Declaration of competing interest

The authors declare no competing interests.
